# Effect of integrated treatment on enhancing the enzymatic hydrolysis of cocksfoot grass and the structural characteristics of co-produced hemicelluloses

**DOI:** 10.1186/s13068-021-01944-8

**Published:** 2021-04-07

**Authors:** Shao-Chao Sun, Dan Sun, Xue-Fei Cao

**Affiliations:** Beijing Key Laboratory of Lignocellulosic Chemistry, Beijing Forestry University, Beijing, 100083 China

**Keywords:** Cocksfoot grass, Hydrothermal pretreatment, Alkali extraction, Enzymatic hydrolysis, Hemicelluloses, Structural characterization

## Abstract

**Background:**

Cocksfoot grass (*Dactylis glomerata* L.) with high biomass yield and rich cellulose can be used to produce bioethanol as fuel additive. In view of this, ultrasonic and hydrothermal pretreatments followed by successive alkali extractions were assembled into an integrated biorefinery process applied on cocksfoot grass to improve its enzymatic hydrolysis. In this work, the effects of ultrasonic and hydrothermal pretreatments followed by sequential alkali extractions on the enzymatic hydrolysis of cocksfoot grass were investigated. In addition, since large amount of hemicelluloses were released during the hydrothermal pretreatment and alkali extraction process, the yields, structural characteristics and differentials of water- and alkali-soluble hemicellulosic fractions isolated from different treatments were also comparatively explored.

**Results:**

The integrated treatment significantly removed amorphous hemicelluloses and lignin, resulting in increased crystallinity of the treated residues. A maximum saccharification rate of 95.1% was obtained from the cellulose-rich substrate after the integrated treatment. In addition, the considerable hemicelluloses (31.4% water-soluble hemicelluloses and 53.4% alkali-soluble hemicelluloses) were isolated during the integrated treatment. The released water-soluble hemicellulosic fractions were found to be more branched as compared with the alkali-soluble hemicellulosic fractions and all hemicellulosic fractions were mixed polysaccharides mainly composed of branched xylans and *β*-glucans.

**Conclusion:**

The combination of ultrasonic and hydrothermal pretreatments followed by successive alkali extractions can dramatically increase the enzymatic saccharification rate of the substrates and produce considerable amounts of hemicelluloses. Detailed information about the enzymatic hydrolysis rates of the treated substrates and the structural characteristics of the co-produced hemicelluloses will help the synergistic utilization of cellulose and hemicellulose in cocksfoot grass.

## Background

Increasing the utilization of agricultural and forestry wastes is conducive to the development of national economy and improvement of global environment. However, similar to other underutilized residues, cocksfoot grass (*Dactylis glomerata* L.) mowed every few months has not been taken seriously as the industrial material [1]. As the excellent forage and green lawn plant, cocksfoot grass has many advantages such as simple harvesting, environmental tolerance, high calorific value (20 kJ/kg), and high yield (20 tons per hectare), which is helpful to obtain additional economic benefits [2]. Therefore, the value-added utilization of cocksfoot grass should be paid more attention. Carbohydrates (cellulose and hemicelluloses) and lignin are the main constituents of cocksfoot grass, among which cellulose as a polymer of glucose is generally used in paper industry or hydrolyzed to produce bioethanol as fuel additive [3, 4]. Hemicelluloses with branched chains are heterogeneous polymers composed of various pentoses, hexoses, and uronic acids, which can be used as feedstocks to prepare various functionalized materials and chemicals [5, 6]. For instance, hemicelluloses have been used to produce xylo-oligosaccharides (XOS) by partial hydrolysis, which is considered as an alternative of antibiotics and can be widely used in health products and feed antibiotics to promote the growth of human intestinal bacteria and alleviate the serious consequences of antibiotic abuse [7]. With the benefit of a series of attractive characteristics and rich carbohydrate reserves, the cocksfoot grass wastes can be developed as useful industrial feedstock to gain additional economic benefits.

Generally, lignocellulosic biomass is recalcitrant to enzymatic and microbial hydrolysis because of the rigid and compact structure of plant cell walls [8]. The heterogeneous complexity and spatial interconnections of these main polymers through covalent or non-covalent bonds in cell walls constitute physical and chemical barriers against enzymes accessibility to the cellulose surface, resulting in a relatively lower digestibility of lignocellulosic biomass [9]. Previous works showed that the highly crystalline structure of cellulose and the existences of hemicelluloses and lignin are the vital factors limiting the enzyme hydrolysis of natural biomass [10, 11]. However, it was also reported that the crystallinity of cellulose was not as important as the ultrastructural changes caused by the removal of components from the tightly packed regions of the cell wall on improving the digestibility of biomass [12]. In view of this, some pretreatment technologies are recommended to facilitate the access of enzymes to cellulose by releasing hemicelluloses and lignin for achieving a maximum yield of fermentable sugar from cellulose [13, 14]. Among various pretreatment methods, hydrothermal pretreatment as an eco-friendly green processing technology has been widely applied on various lignocellulosic biomass to improve their enzymatic digestibility because of its high efficiency on selective removal of hemicelluloses from lignocellulosic materials [15]. It was reported that ultrasound can also be used to pretreat biomass because the ultrasound is capable of decomposing water molecules into free radicals to destroy the network between xylan and lignin [16]. However, most lignin still remains in the plant cell wall after the ultrasound and hydrothermal pretreatment (especially at low temperature) [17]. Therefore, aqueous alkali treatment is generally required to further improve the removal of lignin and residual hemicelluloses since the cleavage of *α*-ether linkages between lignin and hemicelluloses, swelling of cellulose, and fragmentation of lignin usually take place under alkali conditions [18, 19].

In this work, an integrated treatment based on ultrasonic and hydrothermal pretreatments followed by sequential alkali extractions was proposed to improve the enzymatic hydrolysis of cocksfoot grass. The chemical compositions, crystallinity, and saccharification rate of the residues obtained from various treatments were investigated. In addition, the structural characteristics of the co-produced hemicellulosic fractions were also comparatively explored since large amount of hemicelluloses were released during the hydrothermal pretreatment and alkali extraction process. The structure interpretation of the hemicelluloses is meaningful for their wide application as potential bio-based materials.

## Results and discussion

### Chemical compositions of the obtained residues

**Fig. 1:**
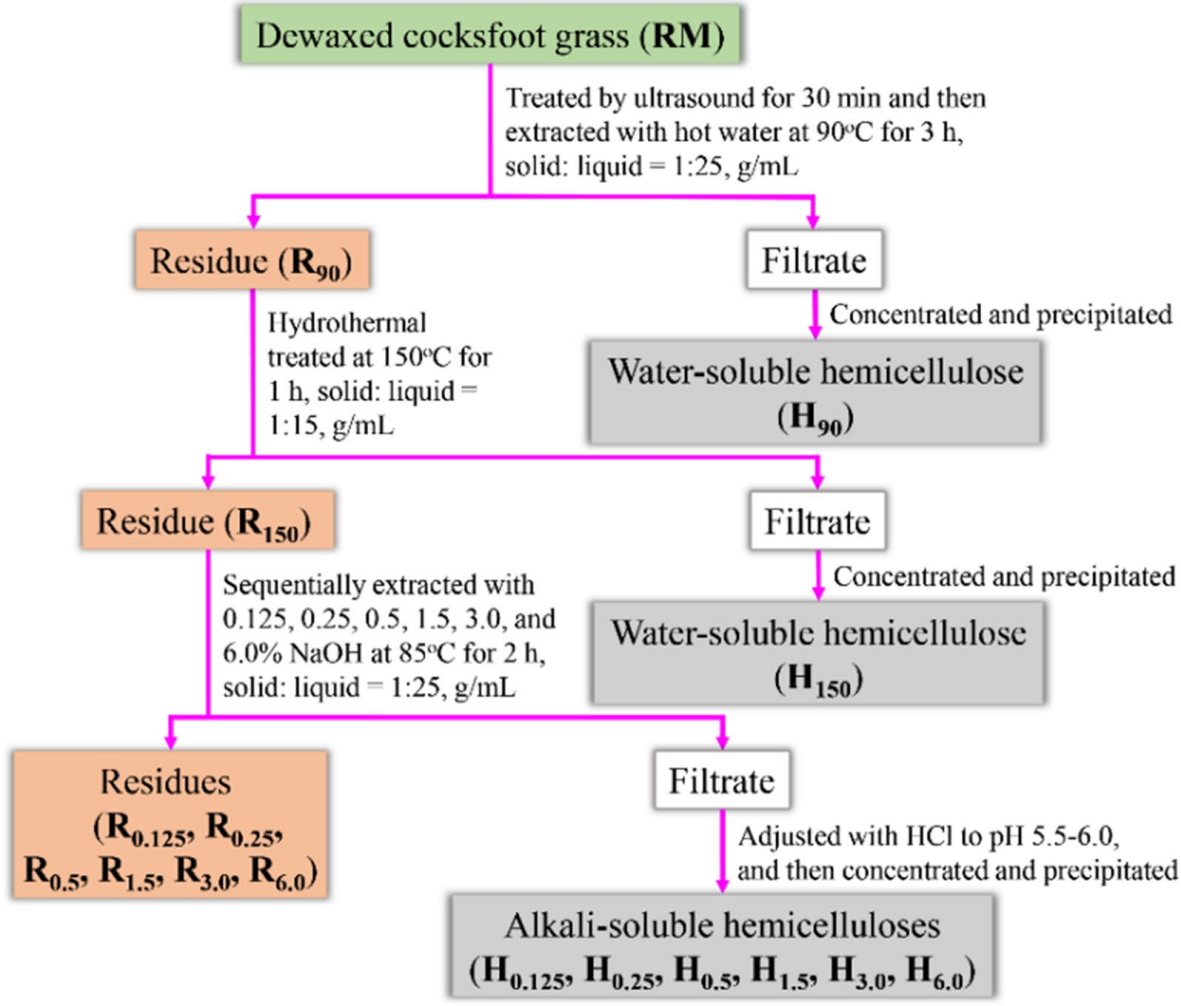
Schematic illustration of the integrated biorefinery process

Ultrasonic and hydrothermal pretreatments followed by sequential alkali extractions were assembled to enhance the enzymatic digestibility of cocksfoot grass and the scheme is exhibited in Fig. 1. The chemical compositions of the dewaxed raw material (RM) and these obtained residues (R_90_, R_150_, R_0.125_, R_0.25_, R_0.5_, R_1.5_, R_3.0_, and R_6.0_) are exhibited in Table 1. As compared with RM, relatively higher glucan (39.6%) and lower xylan (14.4%) were obtained in R_90_, which suggested that partial hemicelluloses were dissolved from the raw material during the ultrasound and hot water extraction process. In addition, hydrothermal pretreatment is usually used to remove hemicelluloses from lignocellulosic materials. As expected, the hemicelluloses were further released, and the cellulose content of R_150_ increased significantly from 39.6 to 45.6%. It seems that the pretreatments executed in this study had no obvious effect on delignification as compared with hemicelluloses, which may be due to the links (mainly ether bonds) between lignin monolignols are not very sensitive to this pretreated condition. During the sequential alkali extraction process, the relative content of hemicelluloses gradually decreased from 17.7 to 5.7% with the increment of the alkali extraction concentration from 0.125 to 6.0%. In addition, lignin, another important inhibitor for enzymatic hydrolysis of cellulose, was largely dissolved during the sequential alkali extractions. Due to the significant removal of hemicelluloses and lignin, the highest content of cellulose (63.6%) and lowest contents of hemicelluloses and lignin (5.7 and 11.1%) were observed in R_6.0_. Nevertheless, small amounts of xylans were still retained in R_6.0_, which indicated that the hemicelluloses closely combined with lignin and cellulose in cell walls were difficult to be completely liberated. Meanwhile, previous work showed that the extensive removal of hemicelluloses will lead to the reassembly of highly crystalline cellulose fibrils, so the retention of small amounts of hemicelluloses is beneficial to the digestion of cellulose [20].

**Table 1:** Chemical compositions of the dewaxed cocksfoot grass and variously treated residues

Samples	Yield^a^ (%)	Chemical compositions^b^ (%)								
		Ara	Gal	Glc	Xyl	Man	GalA	GlcA	AIL	ASL
RM	100.0	7.6	2.4	38.7	15.5	0.2	0.9	0.6	13.0	2.2
R_90_	72.3	5.1	1.8	39.6	14.4	0.1	1.2	0.3	18.3	1.9
R_150_	61.1	1.3	0.8	45.6	12.5	0.2	0.3	0.1	18.0	1.3
R_0.125_	44.9	1.4	0.7	49.4	15.3	0.2	ND	0.1	23.3	1.3
R_0.25_	38.7	0.9	0.4	50.4	12.3	0.3	ND	0.1	20.0	1.5
R_0.5_	35.0	0.8	0.3	55.3	11.5	0.3	ND	0.1	16.7	1.7
R_1.5_	32.3	0.6	0.2	59.5	10.4	0.3	ND	ND	13.3	1.8
R_3.0_	30.9	0.3	0.2	61.5	6.7	0.4	ND	ND	10.0	2.1
R_6.0_	23.3	0.2	0.1	63.6	5.0	0.4	ND	ND	9.6	1.5

^a^Represent the yield of the solid residues $$\begin{gathered} \left( {{\text{the weight of the}}\;{\text{variously}}\;{\text{treated}}\;{\text{cocksfoot }}\;{\text{grass}}} \right)/ \hfill \\ \left( {{\text{the weight of the}}\;{\text{dewaxed}}\;{\text{cocksfoot}}\;{\text{ grass}}} \right)\; \times 100\% \hfill \\ \end{gathered}$$

^b^*Ara* araban, *Gal* galactan, *Glc* glucan, *Xyl* xylan, *Man* mannan, *GalA* galacturonic acid, *GlcA* glucuronic acid, *AIL* acid-insoluble lignin, *ASL* acid-soluble lignin, *ND* not detected

### CP/MAS ^13^C NMR and XRD analysis of the obtained residues

**Fig. 2:**
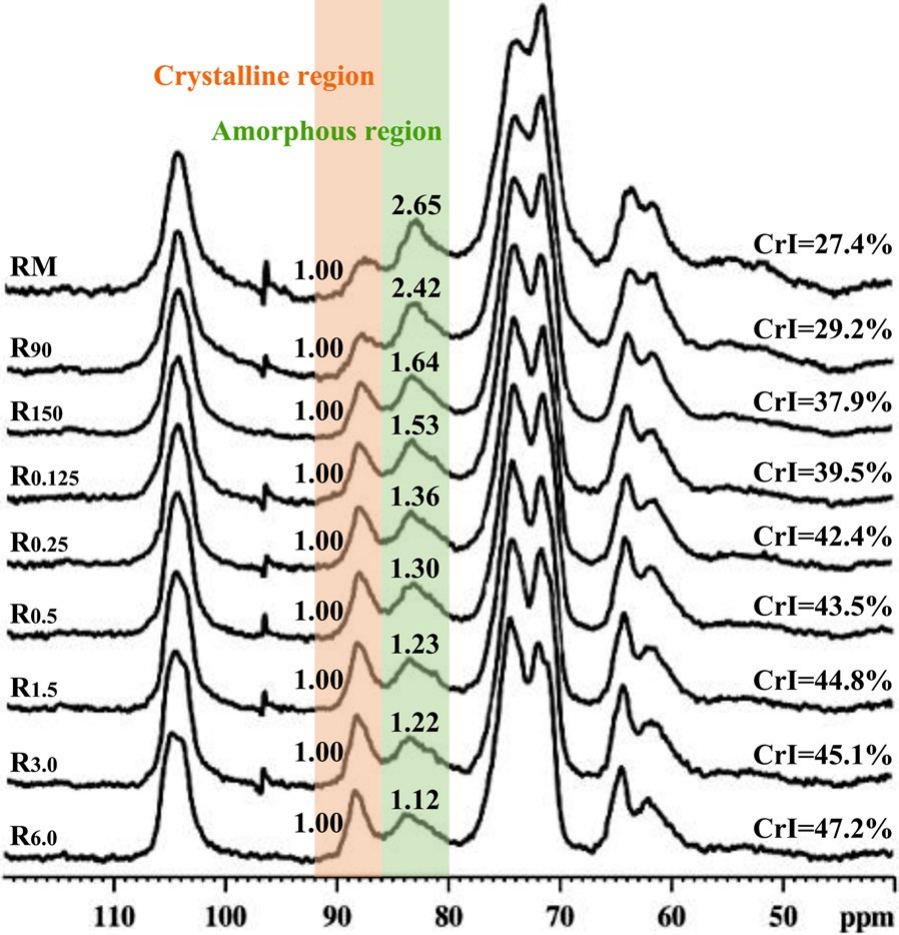
CP/MAS ^13^C NMR spectra of the dewaxed cocksfoot grass and variously treated residues

**Fig. 3:**
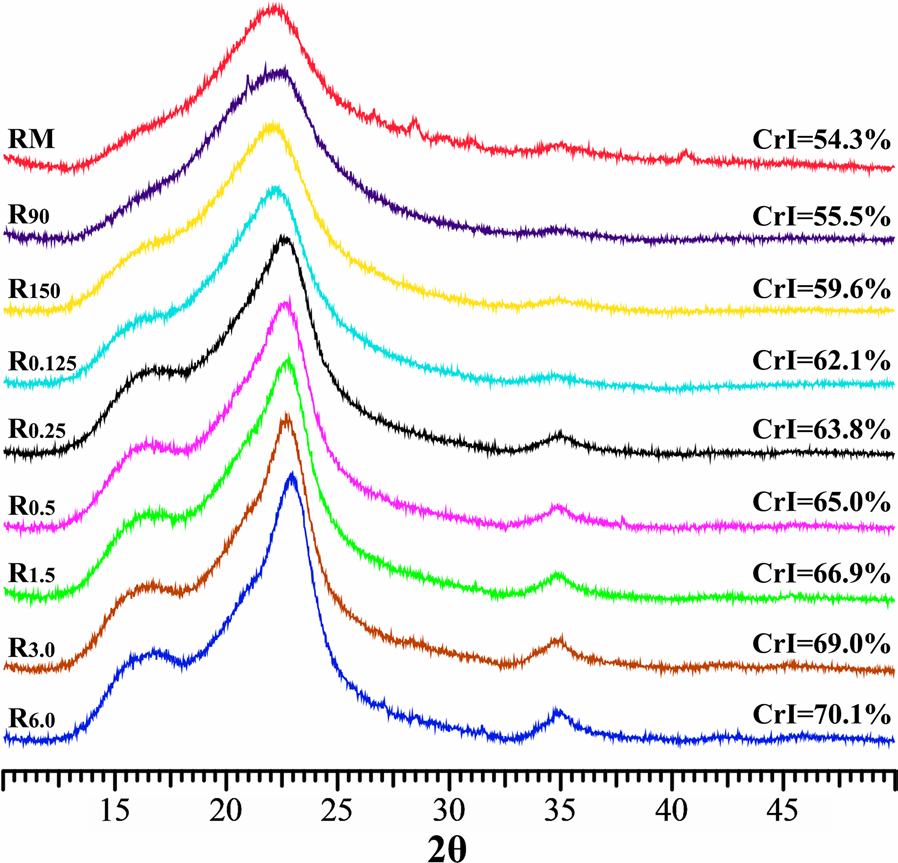
XRD patterns of the dewaxed cocksfoot grass and variously treated residues

Crystallinity is one of the important factors for the saccharification of cellulose-rich residue [12, 21]. To investigate the crystallinity changes of the residues after treatments, solid-state cross-polarization/magic angle spinning (CP/MAS) ^13^C NMR and X-ray diffraction (XRD) were used to analyze the crystallinity indexes (CrI) of the raw material and the variously treated residues. The obtained CP/MAS ^13^C NMR spectra of these samples are shown in Fig. 2. The corresponding crystallinity index (CrI) was calculated by the ratio of the integral value between 86 and 92 ppm to that between 80 and 92 ppm according to previous report [22]. In the region of 80–92 ppm, the signals at 88.3 ppm are assigned to the C4 carbons of the ordered cellulose structure, while the peaks at 83.9 ppm are originated from the C4 carbons of the disordered cellulose. It should be noted that the lignin side chain and hemicelluloses also give signals in the 80–92 ppm range but are weighted towards 80–86 ppm [23]. Therefore, the CrI measured by CP/MAS ^13^C NMR is not only related to the changes of the cellulose crystallinity, but also to the removal of amorphous hemicelluloses and lignin, which can be used as an indicator of overall biomass crystallinity. It can be seen from Fig. 2 that the CrI value of RM was only 27.4%, while the CrI values of R_90_ and R_150_ were up to 29.2 and 37.9%, respectively. Moreover, the CrI values of these hydrothermally treated samples further increased from 39.5 to 47.2% after the following alkali extractions. However, the CrI changes observed in this work were primarily due to the removal of amorphous hemicelluloses and lignin since relatively mild pretreatment condition was used. The XRD patterns of the RM and the treated residues are illustrated in Fig. 3. A typical XRD pattern of cellulose I was observed in all samples. The peaks at 2θ = 14.8°, 16.4°, 22.5°, and 34.7° are assigned to the (1ī0), (110), (200), and (004) diffraction planes of cellulose I, respectively [24]. As compared with the CrI of RM (54.3%), the CrIs of R_90_ and R_150_ slightly increased to 55.5 and 59.6%, respectively. After further treated the hydrothermal residue (R_150_) with 0.125–6.0% alkali solution, the CrI gradually increased from 62.1 to 70.1%. Similar trends were observed from the CP/MAS ^13^C NMR and XRD results.

### Enzymatic saccharification of the obtained residues

**Fig. 4:**
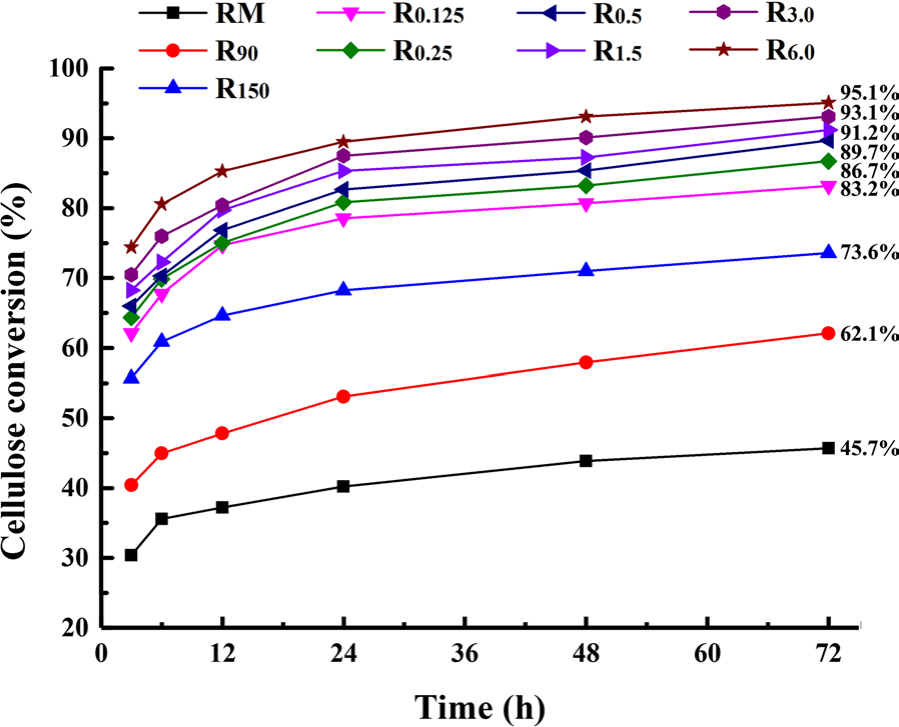
Glucose yield of the dewaxed cocksfoot grass and variously treated residues

Figure 4 shows the cellulose conversion rates of the raw material and variously treated residues after enzymatic hydrolysis. It can be seen that the enzymatic saccharification of these samples was closely related to the extraction conditions. After 72 h enzymolysis, only 45.7% of glucan in the untreated cocksfoot grass was converted into glucose. After the ultrasonic and hydrothermal pretreatments, partial hemicelluloses and lignin were removed from plant cell walls. The enzymatic saccharification rates of the pretreated substrates R_90_ and R_150_ reached 62.1 and 73.6%, respectively. After the successive alkali extractions, the enzymatic hydrolysis rates of these cellulose-rich fractions (R_0.15_, R_0.25_, R_0.5_, R_1.5_, R_3.0_, and R_6.0_) gradually improved to 83.2, 86.7, 89.7, 91.2, 93.1, and 95.1%, respectively. It has been reported that the efficient saccharification of cellulose is closely related to its accessible surface area and the effective adsorption of cellulase on cellulose [25, 26]. Therefore, the relatively high enzymatic hydrolysis rates of the alkali treated substrates were ascribed to the adequate exposure of cellulose fibrils followed by effective adsorption of cellulase caused by the effective removal of hemicelluloses and lignin in the sequential alkali extractions. Overall, the integrated treatment method used in this study could effectively destroy the natural recalcitrance of the cocksfoot grass and the highest glucose yield of 95.1% was achieved from the cellulose-rich substrate R_6.0_.

### Fractional yields and sugar compositions of the water- and alkali-soluble hemicelluloses

Since large amount of hemicelluloses were released during the hydrothermal pretreatment and alkali extraction process, the co-produced hemicellulosic fractions were also comparatively explored. In general, the yields and sugar compositions of hemicelluloses vary widely with the treatment method and condition. Results from Table 2 indicated that the water-soluble hemicellulosic fraction H_90_ had a highest yield of 20.2%, which was consisted of arabinose (16.8%), galactose (15.1%), glucose (38.2%), xylose (20.7%), mannose (1.5%), glucuronic acid (3.6%), and galacturonic acid (4.1%). However, much higher content of xylose (49.6%) and lower content of glucose (16.5%) were found in the water-soluble hemicellulosic fraction H_150_ (11.2%). This fact revealed that the two water-soluble hemicellulosic fractions were mixed polysaccharides mainly composed of branched xylans and glucans. More importantly, the H_90_ released at a relatively lower temperature was higher branched than H_150_ released at a relatively higher temperature. The high content of glucose in water-soluble hemicellulosic fractions (especially H_90_) may also be partially derived from the hydrolysis of xyloglucan [27]. Subsequently, sequential alkali extractions were carried out to improve the removal of non-cellulosic components. As shown in Table 2, the total yields of the six alkali-soluble hemicellulosic fractions accounted for 53.4% of the original hemicelluloses in the dewaxed cocksfoot grass (RM). With the progress of sequential alkali extractions, the yields of alkali-soluble hemicellulosic fractions gradually decreased. The xylose (58.8–72.0%) was the primary sugar constituent of all alkali-soluble hemicellulosic fractions, and its content increased as the NaOH concentration raised from 0.125 to 6.0%. In addition, noticeable amounts of arabinose (8.2–17.9%), glucose (7.1–18.2%), and galactose (1.8–10.9%) together with less amounts of galacturonic acid (0.3–3.4%) and glucuronic acid (0.1–1.8%) were also identified. These results showed that all the alkali-soluble hemicellulosic fractions were mainly composed of branched xylans and glucans similar to the water-soluble hemicelluloses. The difference is that the alkali-soluble hemicellulosic fractions were more linear than that of the water-soluble hemicellulosic fractions. For the branched xylans, the backbone of xylan was substituted by other monosaccharides and uronic acids. Therefore, glucuronoarabinoxylans was the main structural model of all hemicellulosic fractions. The galactose detected was probably resulted from the arabinogalactans or/and galactoarabinoxylans [28]. For the alkali-soluble hemicelluloses, the branch-rich hemicellulosic fractions were liable to be released during the mild alkali extraction process, while the hemicellulosic fractions with more linear structures were easily extracted in the relatively high alkali concentration, which could be reflected by the ratio of arabinose or glucuronic acid to xylose.

**Table 2:** Yields and sugar compositions of the water- and alkali-soluble hemicellulosic fractions

Samples	Yield^a^ (%)	Sugar compositions^b^ (%)						
		Ara	Gal	Glc	Xyl	Man	GlcA	GalA
H_90_	20.2	16.8	15.1	38.2	20.7	1.5	3.6	4.1
H_150_	11.2	16.0	12.8	16.5	49.6	0.9	2.4	2.7
H_0.125_	13.6	17.9	10.9	7.1	58.8	ND	1.8	3.4
H_0.25_	12.3	15.5	8.1	11.3	63.1	ND	1.2	0.8
H_0.5_	9.6	14.1	5.2	16.0	63.3	ND	0.8	0.6
H_1.5_	8.1	8.2	3.4	18.2	66.3	2.4	0.7	0.8
H_3.0_	5.2	9.0	2.0	16.2	71.3	0.8	0.1	0.6
H_6.0_	4.6	9.9	1.8	14.9	72.0	0.9	0.2	0.3

^a^Represent the yield of hemicellulosic fractions $$\begin{gathered} \left( {{\text{the weight of hemicellulosic fractions isolated by different treatments}}} \right)/ \hfill \\ \left( {{\text{the weight of hemicelluloses in the}}\;{\text{dewaxed}}\;{\text{cocksfoot grass}}} \right)\; \times 100\% \hfill \\ \end{gathered}$$

^b^*Ara* arabinose, *Gal* galactose, *Glc* glucose, *Xyl* xylose, *Man* mannose, *GalA* galacturonic acid, *GlcA* glucuronic acid, *ND* not detected

### Molecular weight of the water- and alkali-soluble hemicelluloses

The weight-average (*M*_w_) and number-average (*M*_n_) molecular weights (g/mol) of the water- and alkali-soluble hemicelluloses were comparatively investigated. As listed in Table 3, the *M*_w_ values of two water-soluble hemicellulosic fractions (H_90_ and H_150_) were 30,300 and 28,200 g/mol, respectively. In comparison, all the alkali-soluble hemicellulosic fractions (H_0.125_–H_6.0_) had a relatively higher *M*_w_ values (34,100–44,400 g/mol). This suggested that the combination of ultrasonic and hydrothermal pretreatments promotes the liberation and dissolution of relatively small molecular water-soluble polysaccharides. In contrast, the hemicellulosic fractions with relatively large molecular weights could be released during the aqueous alkali extraction. Moreover, the molecular weights of the alkali-soluble hemicelluloses increased with the alkali concentration from 0.125 to 0.5%. In contrast, when the concentration of alkali exceeded 0.5%, the *M*_w_ values of H_1.5_, H_3.0_, and H_6.0_ decreased, indicating that the higher concentration of alkali extraction leads to the slight degradation of hemicelluloses.

**Table 3:** Weight-average (*M*_w_) and number-average (*M*_n_) molecular weights, and polydispersity index (*M*_w_/*M*_n_) of the water- and alkali-soluble hemicellulosic fractions

Samples	*M*_w_ (g/mol)	*M*_n_ (g/mol)	*M*_w_/*M*_n_
H_90_	30,300	20,000	1.52
H_150_	28,200	19,900	1.42
H_0.125_	34,100	13,200	2.58
H_0.25_	34,500	15,500	2.23
H_0.5_	44,400	18,100	2.45
H_1.5_	41,500	25,000	1.66
H_3.0_	39,700	25,200	1.58
H_6.0_	40,900	25,500	1.60

### FT-IR spectral analysis of the water- and alkali-soluble hemicelluloses

**Fig. 5:**
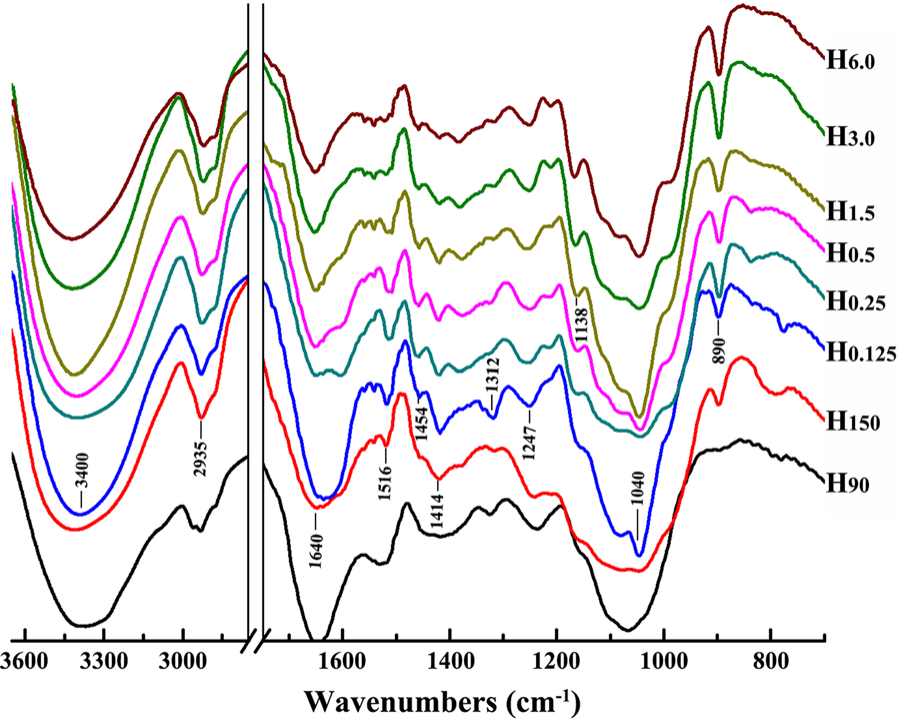
FT-IR spectra of the water- and alkali-soluble hemicellulosic fractions

Fourier transform infrared (FT-IR) spectroscopy can be used for the approximate identification of molecular structures of polysaccharides in plant by combining with other analytical methods. Figure 5 shows the FT-IR spectra of the water- and alkali-soluble hemicellulosic fractions. It can be seen that no significant differences were observed in the spectra of all the samples. The broad peaks at 3400 and 2935 cm^−1^ are ascribed to the O−H stretching vibrations and the C−H stretching vibrations of methyl and methylene of hemicelluloses, respectively. The bands at 1414 cm^−1^ are related to the C−H bending, and the absorption peaks appeared at 1247 cm^−1^ are corresponding to the O−H or C−O bending vibration of typical xylose ring. The major absorption peaks at around 1040 cm^−1^ belong to the C−O−C stretching of glycosidic linkages in xylans. The characteristic bands at 890 cm^−1^ are assigned to the ring frequency or C_1_−H frequency of *β*-glycosidic bonds in hemicelluloses macromolecules [29]. These signals suggested that all the hemicelluloses isolated from cocksfoot grass are typical xylans linked by *β*-1,4 glycosidic bonds. In addition, the characteristic peaks observed at 1516 cm^−1^ are originated from the aromatic skeleton vibrations of bound lignin.

### NMR spectral analysis of the water- and alkali-soluble hemicelluloses

**Fig. 6:**
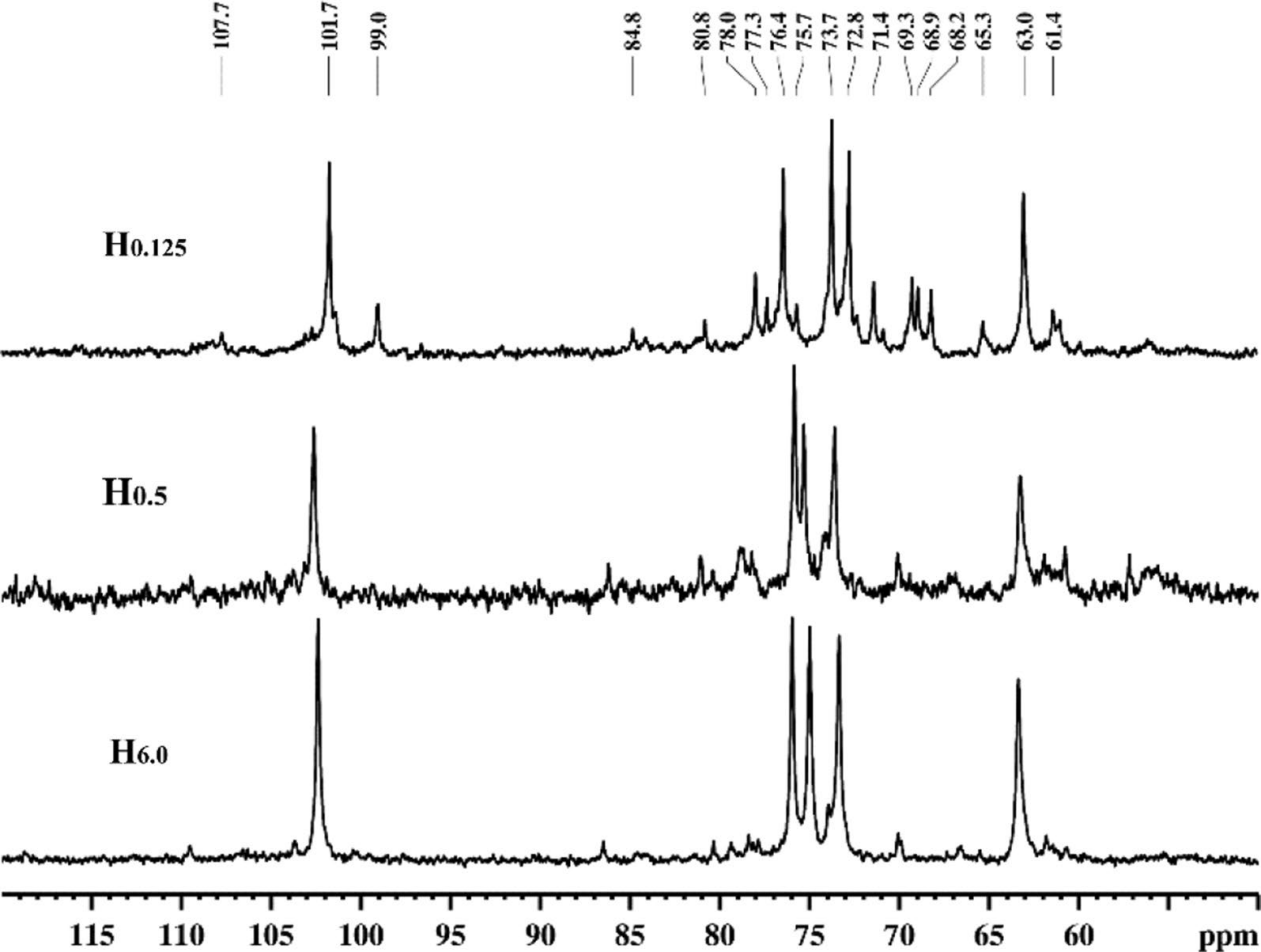
^13^C NMR spectra of the alkali-soluble hemicellulosic fractions (H_0.125_, H_0.5_, and H_6.0_)

**Fig. 7:**
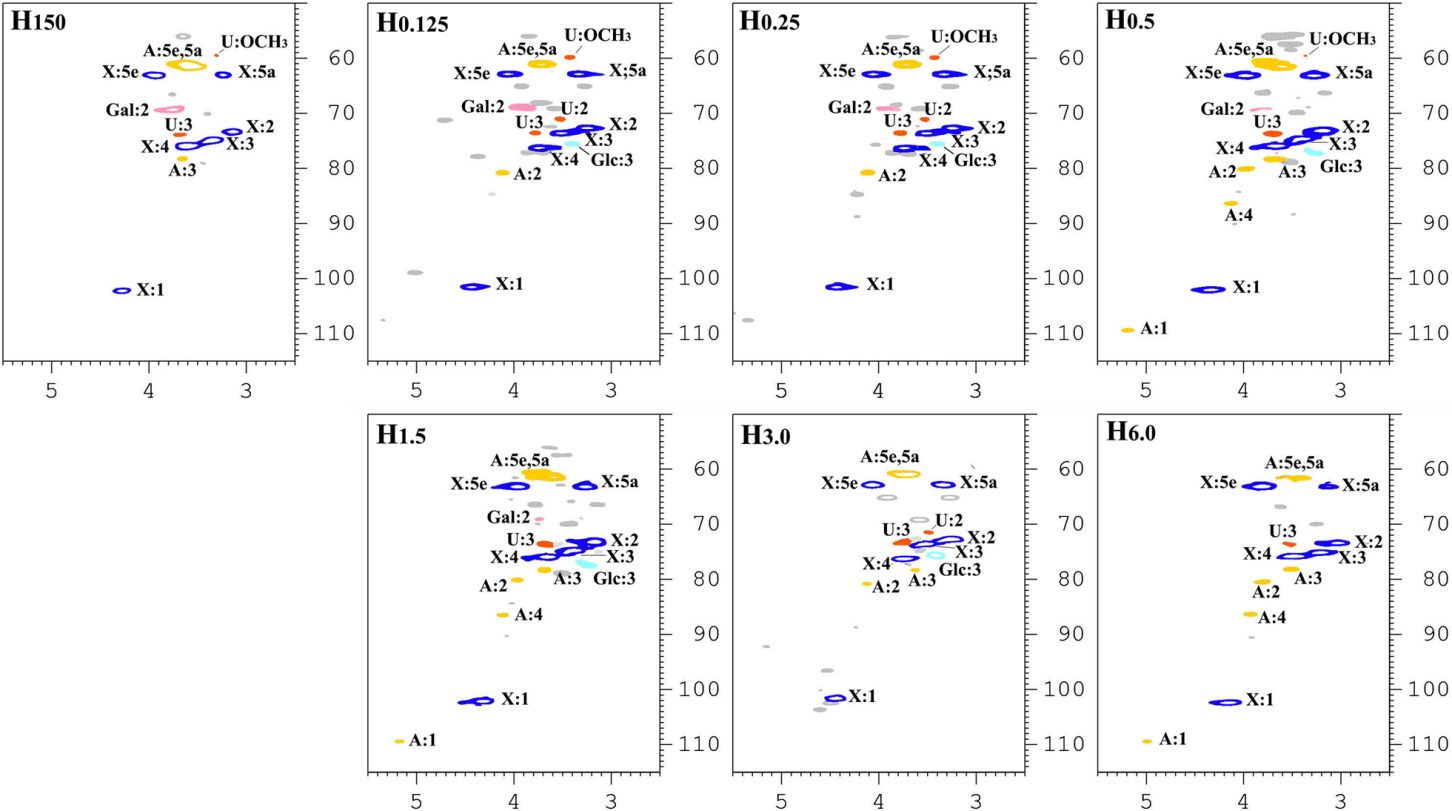
2D-HSQC NMR spectra of the water- and alkali-soluble hemicellulosic fractions. *X*: (1 → 4)-linked-*β*-d-xylopyranosyl units; *U*: 4-*O*-methyl-*α*-d-glucuronic acid units; *A*: *α*-l-arabinofuranosyl units; Gal: galactose units; Glc: glucan units

To further elucidate the exact branching patterns of side-chains attached to the xylan backbone, the water- and alkali-soluble hemicellulosic fractions extracted from cocksfoot grass by different treatments were analyzed by ^13^C and 2D-HSQC NMR techniques. The ^13^C and HSQC NMR spectra obtained are shown in Figs. 6, 7, respectively. All signals in the NMR spectra are assigned and classified in Table 4 based on the previous studies [30–34]. For the ^13^C NMR spectra (Fig. 6) of the three typical alkali-soluble hemicellulosic fractions H_0.125_, H_0.5_, and H_6.0_, the sharp signals located at 101.7, 76.4, 73.7, 72.8, and 63.0 ppm are related to the C-1, C-4, C-3, C-2, and C-5 of *β*-d-xylopyranosyl (*β*-d-Xyl*p*) units, respectively. The signals at 107.7, 84.8, 80.8, and 78.0 ppm are ascribed to the C-1, C-4, C-2, and C-3 of *α*-l-arabinofuranosyl (*α*-l-Ara*f*) units, respectively. The characteristic signal originating from the C-2 of 4-O-methyl-α-d-glucuronic acid (4-*O*-Me-*α*-d-GlcA) units in H_0.125_ spectrum was found at 71.4 ppm.

**Table 4:** Assignments of ^13^C–^1^H cross-signals in HSQC spectra of the water- and alkali-soluble hemicellulosic fractions

Glycosyl^a^	Assignments (ppm)							
		1	2	3	4	5eq^b^	5ax^c^	OCH_3_
X	^13^C	102.2	72.9	74.9	76.2	63.0	63.0	
	^1^H	4.40	3.18	3.40	3.69	3.97	3.26	
U	^13^C		71.1	73.7				59.9
	^1^H		3.50	3.70				3.40
A	^13^C	109.5	80.1	78.3	86.6	61.3	61.3	
	^1^H	5.17	4.10	3.66	4.11	3.71	3.69	
Gal	^13^C		69.0					
	^1^H		3.90					
Glc	^13^C			75.8				
	^1^H			3.39				

^a^*X*, (1 → 4)-linked-*β*-d-xylopyranosyl units; U, 4-*O*-methyl-*α*-d-glucuronic acid units; A, *α*-L-arabinofuranosyl units; Gal, galactose units; Glc, glucan units

^b^eq, equatorial

^c^ax, axial

The detailed structure information of the water- and alkali-soluble hemicellulosic fractions was further clarified by 2D-HSQC NMR technique. As shown in Fig. 7, it was found that the signals observed in the 2D-HSQC NMR spectra of H_150_ are basically consistent with those in alkali-soluble hemicellulosic fractions. The five ^13^C/^1^H cross-signals identified at 102.2/4.40, 76.2/3.69, 74.9/3.40, 72.9/3.18, and 63.0/3.97 and 3.26 ppm are assigned to C_1_–H_1_, C_4_–H_4_, C_3_–H_3_, C_2_–H_2_, and C_5_–H_5_ of (1 → 4)-*β*-d-Xyl*p* backbone, respectively. The two chemical shifts of 3.26 and 3.97 ppm stem from the axial and equatorial protons linked at C-5, respectively. In addition, the correlated cross-peaks corresponding to C_1_–H_1_, C_2_–H_2_, C_4_–H_4_, C_3_–H_3_, and C_5_–H_5_ of α-l-Ara*f* units at *O*-3 are captured at 109.5/5.17, 80.1/4.10, 86.6/4.11, 78.3/3.66, and 61.3/3.71 and 3.69 ppm, respectively. The characteristic signals of C_3_–H_3_, C_2_–H_2_, and –OCH_3_ of 4-*O*-Me-*α*-d-GlcA units at position *O*-2 were found at 73.7/3.70, 71.1/3.50, and 59.9/3.40 ppm, respectively. The signals of C_2_–H_2_ of *α*-galactose units were verified at ^13^C/^1^H of 69.0/3.90 ppm, and the C_3_−H_3_ of *β*-glucans units could be distinguished from the signals at ^13^C/^1^H of 75.8/3.39 ppm. By combining the sugar composition, FT-IR, and NMR data, it was deduced that (1 → 4)-linked *β*-d-Xyl*p* backbone branched with L-Ara*f* units at *O*-2/*O*-3 and 4-*O*-methyl-*α*-d-Glc*p*A units at *O*-2 of the xylose residues is the main chemical structure of all hemicellulosic fractions.

## Conclusions

The combination of ultrasonic and hydrothermal pretreatments followed by successive alkali extractions can dramatically increase the enzymatic saccharification rate of the substrates and produce considerable amounts of hemicelluloses. After the integrated treatment, a maximum glucose yield of 95.1% was obtained from the substrate R_6.0_, which had important reference value for the production of bioethanol from cocksfoot grass. In addition, the water- and alkali-soluble hemicellulosic fractions (84.8%) extracted from different conditions were mainly composed of glucuronoarabinoxylans (i.e*.*, a linear backbone of (1 → 4)-linked *β*-d-Xyl*p* substituted with L-Ara*f* units at *O*-2/*O*-3 and 4-*O*-methyl-*α*-d-Glc*p*A units at *O*-2 of the xylose residues) and *β*-glucans. Moreover, the water-soluble hemicelluloses (31.4%) released at a relatively lower temperature were highly branched than those released at a relatively higher temperature. The alkali-soluble hemicellulosic fractions (53.4%) were more linear than the water-soluble hemicelluloses.

## Methods

### Materials used in this study

Cocksfoot grass (*Dactylis glomerata* L.) of about 30 days was manually harvested from the farm of Beijing Forestry University, China. The dried grass was grinded into small powders, extracted with toluene/ethanol (2:1 v/v) for 5 h, followed by dried at 60 °C for further use. Commercial cellulase was purchased from Novozymes, Beijing, China, with activity of 100 FPU/mL. All other chemical reagents used in this study were analytical grade and used as received.

### Ultrasonic and hydrothermal pretreatments followed by sequential alkali extractions

As illustrated in Fig. 1, the dewaxed raw material (RM) was subjected to an integrated biorefinery process combining ultrasound, hydrothermal pretreatment, and sequential alkali post-extractions. Specifically, the RM was first pretreated by ultrasound radiation at 180 W for 30 min and then extracted with hot water at 90 °C for 3 h at a solid to liquid ratio of 1:25 (g/mL). Afterwards, the solid residue (named as R_90_) obtained after filtration was oven dried at 60 °C and further hydrothermally pretreated at 150 °C for 1 h. After the reaction, the solid product (named as R_150_) was filtered, washed with ultrapure water to neutral, and dried to constant weight. The filtrates obtained from the above pretreatments were concentrated and then added drop by drop to the stirred 95% ethanol (1:3, v/v) to recover the water-soluble hemicelluloses. After centrifugation and freeze-drying, the target water-soluble hemicelluloses were obtained and abbreviated as H_90_ and H_150_, respectively. Then, the R_150_ was further sequentially extracted with 0.125, 0.25, 0.5, 1.5, 3.0, and 6.0% aqueous NaOH at 85 °C for 2 h under a solid to liquid ratio of 1:25 (g/mL). After each extraction, the mixture was filtered and the solid residue was dried and weighted for the next extraction. After the sequential alkali extractions, the obtained solid substrates were labeled as R_0.125_, R_0.25_, R_0.5_, R_1.5_, R_3.0_, and R_6.0_, respectively. Similar to the isolation of the water-soluble hemicellulosic fractions, the alkali-soluble hemicellulosic fractions were recovered from the liquid fractions obtained in each alkali extraction after adjusting to neutral with 6 M HCl. According to the extraction condition used, the isolated alkali-soluble hemicellulosic fractions were denoted as H_0.125_, H_0.25_, H_0.5_, H_1.5_, H_3.0_, and H_6.0_, respectively. All the above experiments were repeated in triplicate.

### Enzymatic hydrolysis

The enzymatic hydrolysis experiments of the dewaxed cocksfoot grass, two pretreated substrates, and six further alkali treated substrates were performed according to the method reported in previous literature [35]. Typically, 0.5 g of sample and 25 mL of 50 mM sodium acetate buffer (pH 4.8) was mixed in a 100 mL Erlenmeyer flask. The enzymatic hydrolysis was carried out at 50 °C for 72 h in an air bath shaking incubator at an enzyme loading of 15 FPU/g substrate. During the enzymatic hydrolysis process, 0.3 mL of hydrolysate was taken out at the time intervals of 3, 6, 12, 24, 48, and 72 h. These hydrolysates were deactivated in boiling water and then analyzed by high-performance anion-exchange chromatography (HPAEC). All enzymatic hydrolysis experiments were conducted three times and the average value was taken.

### Characterization of the obtained residues and isolated hemicelluloses

Chemical compositions (%, w/w) of the dewaxed cocksfoot grass and treated samples were determined according to the National Renewable Energy Laboratory (NREL) standard analytical method [36]. The sugars were measured by HPAEC (Dionex ICS 3000, USA) equipped with a CarboPac™ PA 20 column (3 × 150 mm) and an amperometric detector. The content of acid-insoluble and acid-soluble lignin was obtained by filtration and UV–Vis spectrophotometry, respectively. The crystallinities of the obtained residues were estimated by CP/MAS ^13^C NMR and XRD, respectively. For CP/MAS ^13^C NMR spectra, the dried sample was packed in a 4 mm zirconia (ZrO_2_) rotor and detected using a cross-polarization pulse program with the matching time of 1 ms and the delay of 2 s between transients. XRD patterns of the residues were performed on a Rigaku Ultima IV diffractometer (Japan) with Cu Kα radiation from 10° to 50°. The crystallinity index (CrI) obtained from the XRD data was calculated according to the following formula:

$${\text{CrI }}\left( \% \right) \, = \, \left[ {\left( {I_{{00{2}}} - I_{{{\text{am}}}} } \right)/I_{{00{2}}} } \right] \, \times { 1}00,$$

where *I*_002_ is the maximum intensity of cellulose at about 2θ = 22.5° and *I*_am_ is the minimum intensity from the amorphous phase at approximately 2θ = 18°.

Sugar compositions and molecular weights of water- and alkali-soluble hemicellulosic fractions were determined by HPAEC and gel permeation chromatography (GPC, Agilent 1200, USA), respectively, according to the previous reports [37, 38]. The GPC equipped with a PL aquagel-OH MIXED-M column (300 × 7.5 mm) and a differential refractive index detector. 0.02 M NaCl in 0.005 M sodium phosphate buffer (pH 7.5) was used as eluent. FT-IR spectra of two types of hemicellulosic fractions were recorded on a spectrophotometer using a KBr disk containing 1% finely ground sample. Solution-state NMR spectra of these hemicelluloses were recorded at 25 °C on a Bruker AVIII 400 MHz spectrometer. The hemicellulose samples (20 mg for 2D-HSQC and 60 mg for ^13^C NMR) were dissolved in 0.5 mL of D_2_O. To improve the solubility of hemicelluloses, few drops of sodium deuteroxide (7.5 M NaOD) were added. The number of collected complex points was 1024 for ^1^H-dimension with a recycle delay of 1.5 s. The number of transients was 128, and 256 time increments were recorded in the ^13^C-dimension. For ^13^C NMR, a 30° pulse flipping angle, 9.2 μs pulse width, 1.36 s acquisition time, and 1.89 s relaxation delay time were used.

## Data Availability

All data generated or analyzed during this study are included in this published article.
